# Micron-scale voltage and [Ca^2+^]_i_ imaging in the intact heart

**DOI:** 10.3389/fphys.2014.00451

**Published:** 2014-12-02

**Authors:** Xiao-long Lu, Michael Rubart

**Affiliations:** Riley Heart Research Center, Department of Pediatrics, Wells Center for Pediatric Research, Indiana University School of MedicineIndianapolis, IN, USA

**Keywords:** laser scanning microscopy, optical voltage mapping, optical [Ca^2+^]_i_ mapping, stem cell transplantation, Langendorff-perfused heart

## Abstract

Studies in isolated cardiomyocytes have provided tremendous information at the cellular and molecular level concerning regulation of transmembrane voltage (V_m_) and intracellular calcium ([Ca^2+^]_i_). The ability to use the information gleaned to gain insight into the function of ion channels and Ca^2+^ handling proteins in a more complex system, e.g., the intact heart, has remained a challenge. We have developed laser scanning fluorescence microscopy-based approaches to monitor, at the sub-cellular to multi-cellular level in the immobilized, Langendorff-perfused mouse heart, dynamic changes in [Ca^2+^]_i_ and V_m_. This article will review the use of single- or dual-photon laser scanning microscopy [Ca^2+^]_i_ imaging in conjunction with transgenic reporter technology to (a) interrogate the extent to which transplanted, donor-derived myocytes or cardiac stem cell-derived *de novo* myocytes are capable of forming a functional syncytium with the pre-existing myocardium, using entrainment of [Ca^2+^]_i_ transients by the electrical activity of the recipient heart as a surrogate for electrical coupling, and (b) characterize the Ca^2+^ handling phenotypes of cellular implants. Further, we will review the ability of laser scanning fluorescence microscopy in conjunction with a fast-response voltage-sensitive to resolve, on a subcellular level in Langendorff-perfused mouse hearts, V_m_ dynamics that typically occur during the course of a cardiac action potential. Specifically, the utility of this technique to measure microscopic-scale voltage gradients in the normal and diseased heart is discussed.

## Introduction

Studies in single isolated cardiomyocytes have provided important information at the cellular and molecular level concerning the electrical properties and Ca^2+^ regulation. Because isolated myocytes are disconnected from their neighboring myocytes as well as their non-myocytes (which are important modulators of cardiomyocyte function), this profoundly impacts on fundament physiological properties. For example, cardiomyocyte action potential properties are very likely to be different in intact muscle compared to single cells. The peak of the action potential is typically more positive in the isolated myocyte, because there is less passive outward current to oppose the depolarizing effect of locally activating inward sodium current. Multicellular preparations may thus be valuable for electrophysiological characterizations, specifically those related to action potential properties and propagation. In addition, such preparations would enable the study of key issues about myocyte Ca^2+^ regulation, including the conditions required for Ca^2+^ wave initiation and propagation from cell-to-cell. Overall, cardiac physiology/pathophysiology mandates the development of assays capable of resolving dynamic events with cellular/subcellular resolution in intact cardiac tissue.

In their pioneering study, Wier and co-workers developed a confocal laser scanning microscopy-based technique to monitor local sarcoplasmic reticulum (SR) Ca^2+^ release phenomena and the propagation of Ca^2+^ waves in isolated rat papillary muscles iontophoretically loaded with the calcium indicator fluo-3 (Wier et al., 1997). Fujiwara and co-workers also used laser scanning microscopy on intact, retrogradely perfused rat hearts loaded with both a calcium- and a voltage-sensitive dye to examine the dynamic interplay between spontaneous, regenerative SR Ca^2+^ release events and arrhythmogenic electrical activity of *in situ* cardiomyocytes (Fujiwara et al., 2008). This review focuses on the ability of single- or dual-photon laser scanning fluorescence microscopy (i) to assess the function of intracardiac cell transplants and stem cell-derived *de novo* myocardium, when used in combination with transgenic reporter technology, and (ii) to measure spatiotemporal dispersion of electrical and Ca^2+^ signals at the microscopic scale in normal and diseased heart.

## Two-photon excitation imaging of intracellular calcium dynamics in langendorff-perfused mouse hearts

**Figure 1:**
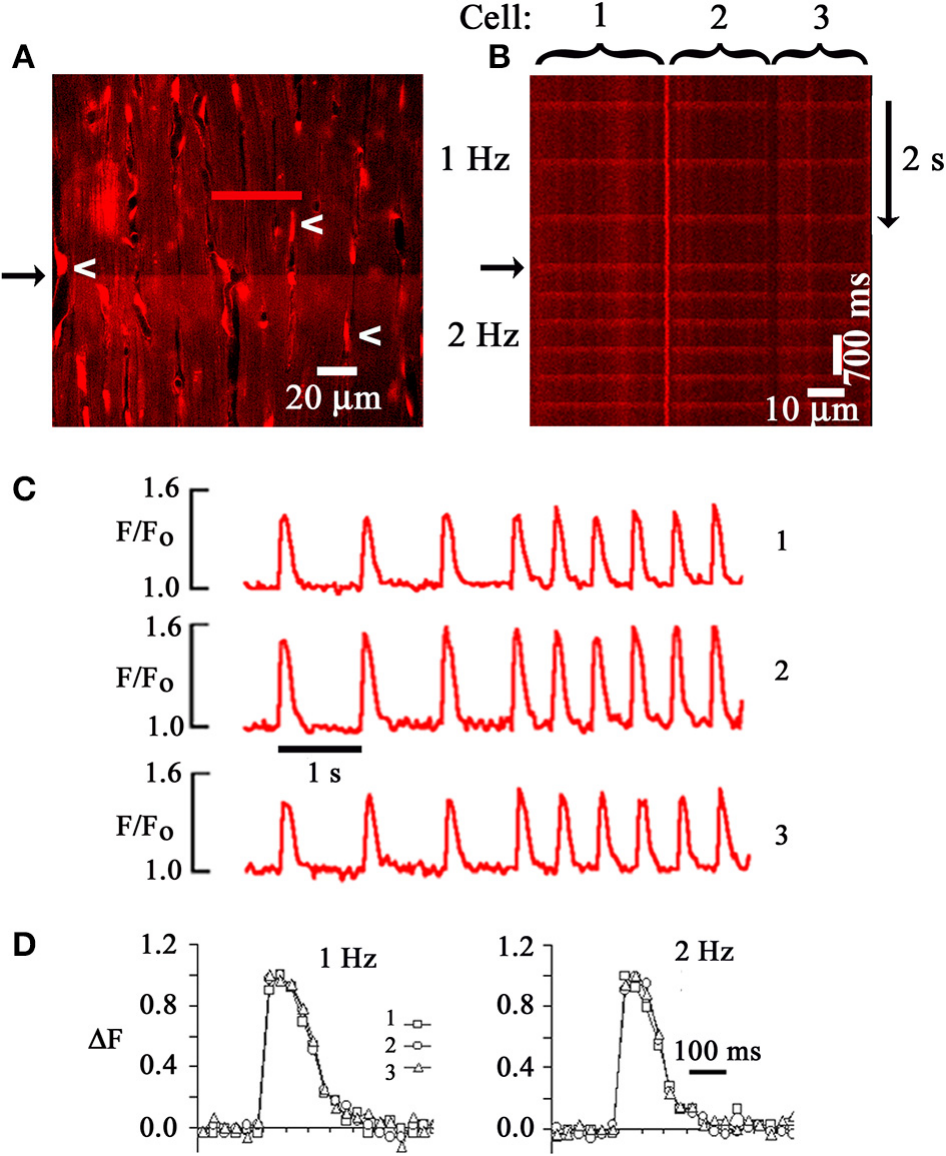
**Two-photon laser scanning microscopy-based imaging of action potential-evoked [Ca^2+^]_i_ transients in the Langendorff-perfused mouse heart. (A)** Frame-mode image of a rhod-2 loaded heart obtained from the left ventricular epicardium during two-photon excitation (810 nm). Emission was measured in the 560–650 nm range. White arrows point to endothelial cell nuclei. Cytochalasin D (50 μmol/L) was used to uncouple contraction from excitation. An electrical stimulus delivered at a point remote from the imaged area caused a simultaneous increase in rhod-2 fluorescence, i.e., [Ca^2+^]_i_, along the horizontal scan line (black arrow). **(B)** Line-scan image obtained by repeatedly scanning along the red line in **(A)** and stacking consecutive line scans vertically. The scan line traversed three neighboring cardiomyocytes. Periodic increases in rhod-2 fluorescence intensity correspond to [Ca^2+^]_i_ transients evoked by propagating action potentials. Black arrow marks increase in pacing rate from 1 to 2 Hz. **(C)** time courses of spatially averaged rhod-2 fluorescence intensities (F) normalized to pre-stimulus F (F_o_) for each of the three juxtaposed cardiomyocytes in **(B)**. **(D)** Superimposed tracings of the changes in [Ca^2+^]_i_ as a function of time for the three cardiomyocytes in **(B)**. Normalized changes in F (ΔF) were obtained by dividing (F-F_0_) at each data point by the maximal (F-F_o_). With permission from the American Physiological Society.

**Figure 2:**
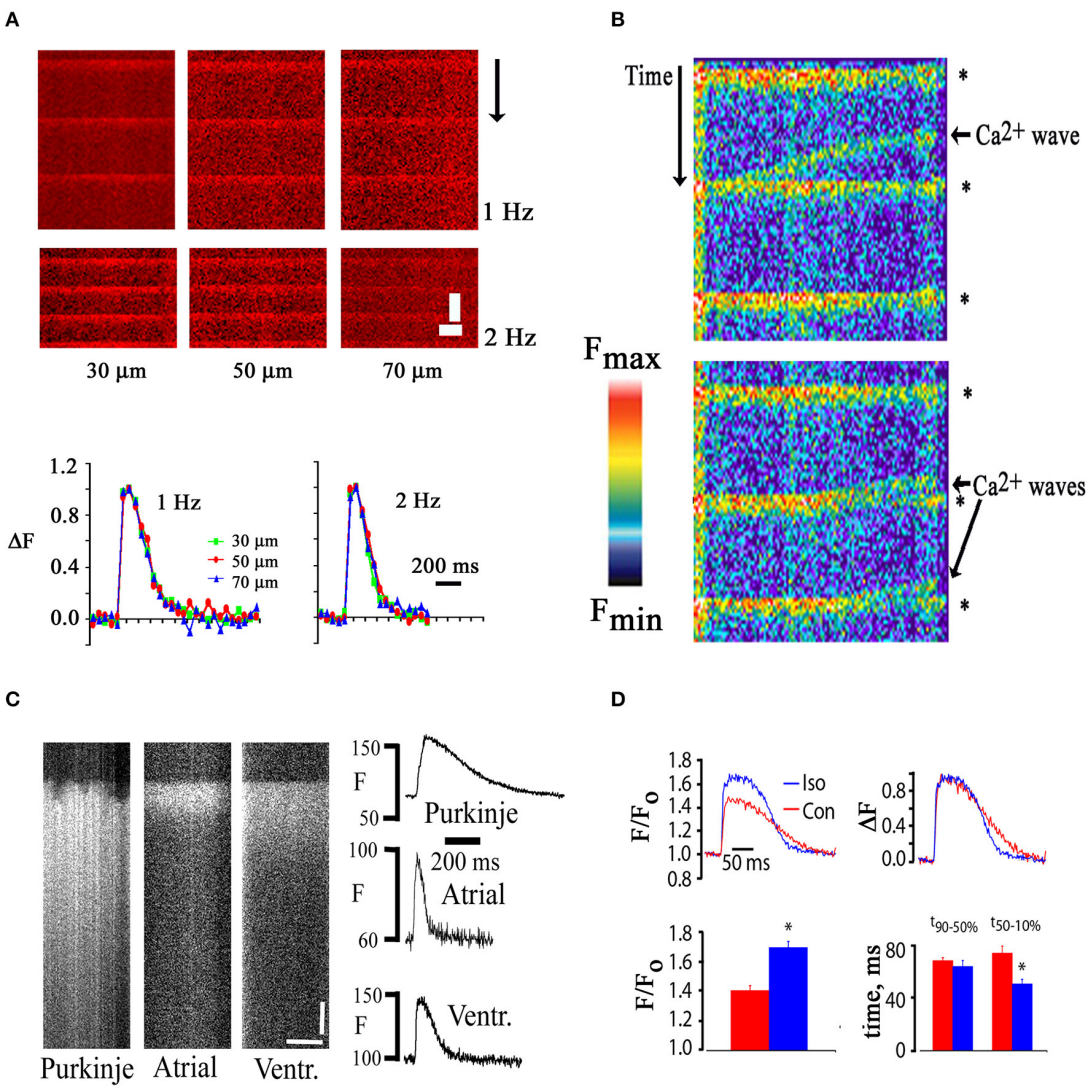
**(A)** Similar time courses of electrically evoked [Ca^2+^]_i_ transients recorded from *in situ* cardiomyocytes located at increasing depth from the epicardial surface. Upper panel, line scan images; lower panel: plots of spatially averaged, normalized changes in rhod-2 fluorescence intensity at the depths indicated as a function of time. Recordings were obtained in the presence of cytochalasin D (50 μmol/L). Scale bars: 500 ms vertically, 5 μm horizontally. **(B)** Line scan image obtained from a rhod-2 loaded mouse heart during remote point electrical pacing at 1 Hz. Rhod-2 fluorescence intensity is shown in pseudocolors, with red and black representing the highest and lowest fluorescence intensity, respectively. Collision of spontaneously arising Ca^2+^ waves with electrical stimulation-evoked [Ca^2+^]_i_ transients (asterisks) caused annihilation of the waves. **(C)** Line-scan images of electrically evoked rhod-2 transients of a right ventricular endocardial Purkinje myocyte, a left atrial myocyte, and a left ventricular mycoyte in Langendorff-perfused mouse hearts. Plots of spatially averaged rhod-2 transients as a function of time for each cell type are shown on the right. Scale bars, 50 ms vertically, 10 μm horizontally. **(D)** Changes in [Ca^2+^]_i_ transient amplitude and duration following exposure of a rhod-2–loaded, isolated perfused mouse heart to isoproterenol (100 nmol/L) in the presence of cytochalasin D (50 μmol/L). Bar graphs show mean changes in peak F/F_o_ (lower left panel) and in the time course of fluorescence recovery (lower right panel). With permission from the American Physiological Society.

We previously developed a technique to optically monitor, on a sub- to multi-cellular scale, intracellular calcium ([Ca^2+^]_i_) dynamics in the intact, Langendorff-perfused mouse heart, using two-photon laser scanning microscopy (TPLSM) in conjunction with calcium-sensitive fluorescent dyes (Rubart et al., 2003). Two-photon molecular excitation-based microscopy offers advantages over traditional confocal approaches in that it permits acquisition of fluorescence signals originating deep within strongly light-scattering biological specimens. This is accomplished by illuminating the tissue with ultrafast pulsed (80 MHz), long wavelength (>700 nm) laser light, resulting in a high photon density in the diffraction-limited volume around the focal point of the objective lens. Because lower energy photons are used, fluorophore emission occurs only following excitation by two or more photons. Since the probability of two photon excitation declines with the fourth power of distance from the focal point, fluorophore excitation (and thus emission) is confined to an extremely thin optical section, giving two-photon excitation microscopy its intrinsic 3D resolution. The magnitude of the two-photon excitation volume is primarily determined by the numerical aperture of the objective lens and the excitation wavelength, and can be as small as ~1 μm^3^ for a 1.2 numerical aperture lens and 800-nm light, with less than 1 μm axial extension. The excitation volume increases with longer wavelength light and lower numerical aperture. Because two-photon excitation imaging does not suffer from degradation of signal-to-noise ratio to nearly the same extent as confocal imaging, it can provide high-contrast images even at significant depths (>50 μm) in strongly scattering tissue (Centonze and White, 1998; Rubart, 2004). We used two-photon excitation in conjunction with scanning microscopy to measure [Ca^2+^]_i_ -dependent changes in intensity of the fluorescent calcium indicator rhod-2 at the single cardiomyocyte level in a buffer-perfused mouse heart preparation in the presence of the excitation-contraction uncoupler cytochalasin D. Representative results are summarized in Figure 1. Figure 1A illustrates the distribution of rhod-2 fluorescence in a 220 × 220 μm^2^ portion of left ventricular epicardium during remote electrical stimulation. Rhod-2 fluorescence intensity rises simultaneously in all cardiomyocytes along the horizontal scan line (black arrow), indicating that SR Ca^2+^ release activated by propagating depolarization is highly synchronized among neighboring cardiomyocytes *in situ*, at least within the temporal resolution of the system (line-scan speed = 1.33 ms/line). The line-scan image (Figure 1B), which was obtained by repeatedly scanning along a line encompassing three juxtaposed cardiomyocytes in transverse direction and stacking all consecutive line-scans vertically, reveals action potential-evoked, periodic rhod-2 transients occurring simultaneously in all three cells. Plots of spatially averaged, normalized rhod-2 fluorescence intensities (F/F_o_) as a function of time confirm entrainment of [Ca^2+^]_i_ transients in all three cardiomyocytes by stimulus-evoked propagating action potentials. Further, superimposition of normalized [Ca^2+^]_i_ transients reveal acceleration of [Ca^2+^]_i_ decay kinetics with an increase in pacing rate (Figure 1D), as well as similar time courses for both [Ca^2+^]_i_ rise and decline in the juxtaposed cardiomyocytes. Line-scan mode images of action potential-evoked rhod-2 transients obtained at increasing distance from the left epicardial surface demonstrated that the kinetics of normalized fluorescent transients are essentially superimposable (Figure 2A). The latter observation is in agreement with a recent study by Ghouri et al. (2013), who were able to resolve action potential-evoked [Ca^2+^]_i_ transients up to a depth of ~400 μm in Langendorff-perfused, immobilized rat hearts, using the ratiometric Ca^2+^-sensitive fura-2. Together, these results support the notion that the processes underlying excitation-induced Ca^2+^ release from the SR and ensuing removal of Ca^2+^ from the cytosol are highly coordinated among neighboring cardiomyocytes *in situ*.

Occasionally, propagating [Ca^2+^]_i_ elevations (“Ca waves”) were observed to occur between stimulated [Ca^2+^]_i_ transients as shown in Figure 2B. Rhod-2 fluorescence transients evoked by electrical stimulation truncated calcium waves which is consistent with the notion of a preferential cytosolic origin of the dynamic rhod-2 signal. Ca^2+^ waves have previously been shown to result from propagating activation of neighboring clusters of ryanodine receptors in the SR membrane. Annihilation of Ca^2+^ waves by colliding, action potential-induced transients is thought to be due to ryanodine receptor refractoriness in the wake of a wave of calcium release (Bers, 2002). Such interaction would not be expected to occur if the electrically evoked increase in indicator fluorescence were confined to, e.g., mitochondria, or other organelles. These observations are in agreement with results of a previous study by Del Nido and co-workers, who found no indication of mitochondrial deposition of rhod-2/AM in isolated guinea-pig hearts (Del Nido et al., 1998).

TPLSM imaging also revealed distinct differences in the spatiotemporal profiles of action potential-evoked [Ca^2+^]_i_ transients in atrial, ventricular and Purkinje myocytes as shown in Figure 2C. Purkinje myocyte transients were recorded from the right side of the interventricular septum. In contrast to both the atrial and ventricular transient, the Purkinje transient exhibits large inhomogeneity during the initial rise in [Ca^2+^]_i_. Such degrees of heterogeneity have previously been described for Purkinje myocytes isolated from rabbit hearts (Cordeiro et al., 2001) and have been shown to reflect inhomogeneity of SR Ca^2+^ release, resulting from a poorly developed or lack of a t-tubular system in these cells. The [Ca^2+^]_i_ transient duration is markedly shorter in the atrial myocyte compared to both the Purkinje and ventricular cardiomyocyte.

Finally, we also tested the responsiveness of *in situ* cardiomyocyte Ca^2+^ handling to beta-adrenergic stimulation. We found that the peak [Ca^2+^]_i_ transient amplitude is significantly increased and the [Ca^2+^]_i_ transient decay is significantly accelerated in *in situ* cardiomyocytes upon exposure to isoproterenol (Figure 2D).

The frequency dependence of [Ca^2+^]_i_ transient decay, the [Ca^2+^]_i_ response to beta-adrenergic stimulation, and the spatiotemporal profile of SR Ca^2+^ release (see below) of *in situ* cardiomyocytes in the presence of cytochalasin D are at least qualitatively similar to those seen in isolated mouse ventricular cardiomyocytes in the absence of excitation-contraction uncouplers but under otherwise comparable experimental conditions (temperature, extracellular [Ca^2+^]), supporting the notion that cytochalasin D not only effectively uncouples contraction from excitation but also retains basic properties of action potential-evoked [Ca^2+^]_i_ transients of cardiomyocytes. Previous studies by the Salama laboratory (Baker et al., 2000, 2004) demonstrated that cytochalasin D prolongs mouse ventricular action potential duration in a concentration-dependent manner. Changes in membrane potential would affect the activities of voltage-dependent calcium conductances, leading to changes in the amplitude and/or kinetics of [Ca^2+^]_i_ transients. Use of the alternative excitation-contraction uncoupler 2,3-butanedione monoxime at concentrations that were necessary to eliminate motion (50 mM) is not feasible because of its adverse effects on electrical excitability (Rubart et al., 2003). Blebbistatin has been widely used for optical voltage or [Ca^2+^]_i_ mapping in mouse hearts to suppress motion artifacts, with reportedly no major effects on cardiac electrophysiology (Fedorov et al., 2007). More recently, Kelly et al. were able to demonstrate that blebbistatin at a concentration of 10 μmol/L in combination with low concentrations of 2,3-butanedione monoxime (10 mmol/L) sufficiently suppressed motion artifacts in Langendorff-perfused rabbit hearts during TPLSM V_m_ imaging, without significantly altering action potential upstroke or repolarization (Kelly et al., 2013). The laboratory of Andrew Wasserstrom has used cytochalasin D alone or in combination with 2,3-butanedione monoxime during *in situ* confocal [Ca^2+^]_i_ imaging in Langendorff-perfused rat hearts to elucidate heart rate-dependent heterogeneities in intracellular Ca^2+^ signaling (Aistrup et al., 2006, 2009) as well as the relationship between spontaneous sarcoplasmic Ca^2+^ release events and triggered electrical activity (Wasserstrom et al., 2010). Although cytochalasin D may exert as yet undefined minor effects on the time course and/or magnitude of stimulated [Ca^2+^]_i_ transients, our observations and those by others support the notion that the use of this fungal metabolite does not *a priori* preclude the study of cardiomyocyte Ca^2+^ handling in intact heart.

Overall, the TPLSM-based system permits imaging of [Ca^2+^]_i_dynamics with subcellular resolution in individual *in situ* cardiomyocytes at tissue depths that are considered inaccessible to single-photon laser scanning confocal microscopy. The system can be used to make [Ca^2+^]_i_ measurements at a level that is achievable in single isolated cardiomyocytes.

## TPLSM [Ca^2+^]_i_ imaging for functional assessment of intracardiac cell transplants

**Figure 3:**
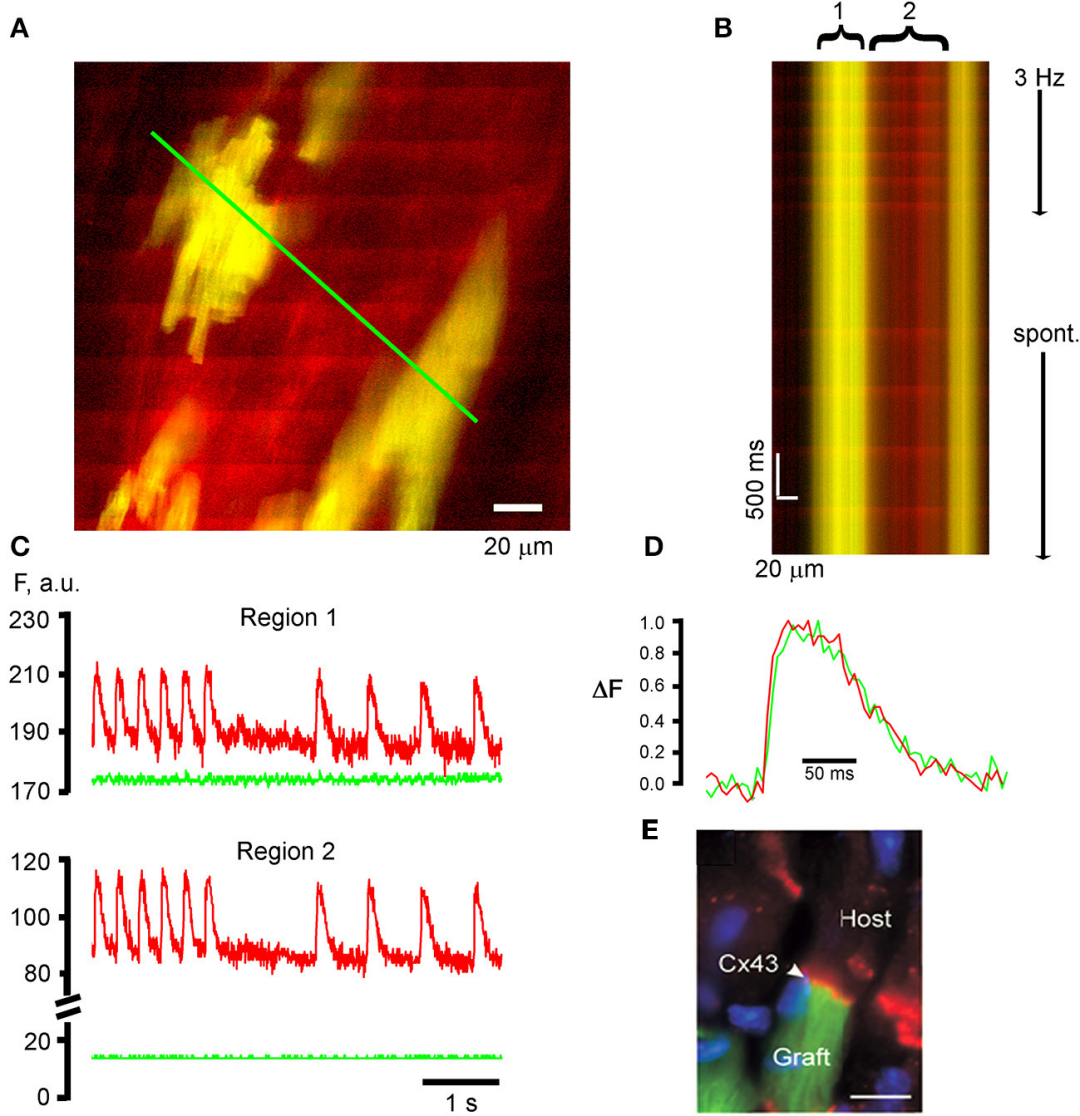
**TPLSM [Ca^2+^]_i_ imaging for functional assessment of intracardiac donor cell transplants. (A)** PSC-derived cardiomyocytes participate in a functional syncytium with the host myocardium following their intracardiac transplantation. The frame mode image was taken from a rhod-2—loaded, Langendorff-perfused mouse heart at 21 days following intracardiac injection of 1,00,000 EGFP-expressing, PSC-derived cardiomyoctyes into a non-expressing mouse heart. Donor myocytes appear yellow due to the overlap of red rhod-2 fluorescence and green EGFP fluorescence. Periodic, ripple-like elevations in rhod-2 fluorescence intensity correspond to remote electrical stimulation (3 Hz)-evoked [Ca^2+^]_i_ transients. **(B)** Line-scan image that was obtained by repeatedly scanning along the green line in **(A)** (500 Hz line-scan rate) and stacking consecutive line-scans vertically. The line encompasses EGFP-expressing, PSC-derived donor myocytes (yellow) and non-expressing host cardiomyocytes (red). **(C)** Plots of spatially averaged rhod-2 and EGFP fluorescence intensities as a function of time for the donor-derived (1) and host (2) cardiomyocytes from the line-scan image in **(B)**. Note the absence of [Ca^2+^]_i_ transient-associated changes in EGFP fluorescence intensity (green traces), indicating effective uncoupling of contraction from excitation by cytochalasin D (50 μmol/L). **(D)** Superimpositon of normalized [Ca^2+^]_i_ transients from the donor and host cardiomyocytes. **(E)** Connexin 43 expression (red signal) at the end-to-end junction between an EGFP-expressing PSC-derived donor myocyte (green) and a non-expressing host cardiomyocyte. Blue signal = nuclear DAPI straining. **(C–E)** with permission from the Journal of Clinical Investigation.

Transplantation of cardiomyocytes or cells with cardiomyogenic potential into the diseased heart constitutes a promising therapy to restore cardiac pump function provided that the *de novo* myocardium functionally integrates into the host tissue, directly contributing to the overall contractile force of the recipient heart (Rubart and Field, 2006). We previously developed a TPLSM-based assay to interrogate the functional fate of transplanted donor cells at the single cell level *in situ*, using entrainment of donor cell [Ca^2+^]_i_ transients by the electrical activity of the surrounding host myocardium as a read out. To be able to unambiguously differentiate donor and host cells in the living heart during optical imaging, we utilized donor cells expressing a transgenic green fluorescent reporter protein. Figure 3 illustrates the ability of our optical assay to monitor the functional fate of donor-derived myocytes, using intracardiac delivery of parthenogenetic stem cell (PSC)-derived cardiomyocytes as an example. In this particular study, cardiomyocytes were generated from a PSC line carrying a transgene composed of sequences encoding the cardiomyocyte-specific α-MHC promoter and the EGFP reporter (MHC-EGFP) (Rubart et al., 2004a), using a recently developed protocol (Didié et al., 2013). PSC-derived MHC-EGFP cardiomyocytes were then injected into adult mouse hearts. Three weeks later, the hearts were subjected to TPLSM [Ca^2+^]_i_imaging as described above. Frame-mode images taken at the graft-host interface during remote electrical point stimulation reveal that action potential—evoked rhod-2 transients occur synchronously in EGFP-expressing, i.e., donor-derived, cardiomyocytes and non-expressing host cardiomyocytes (Figure 3A), indicating that they are functionally coupled with each other. The line-scan image as well as the time-plots of the spatially integrated rhod-2 fluorescence intensities derived from it further demonstrate no detectable delay in the onset of the respective transients during electrical stimulation or sinus rhythm (Figures 3B,C). [Ca^2+^]_i_ transient kinetics were indistinguishable between neighboring donor and host myocytes (Figure 3D). Finally, PSC-myocyte grafts displayed connexin43-positive junctional complexes between engrafted and native cardiomyocytes (Figure 3E). Taken together, the observations that the [Ca^2+^]_i_ transients in PSC-derived donor cells, elicited by normal electrical propagation, show a time course identical to Ca^2+^ transients in the host cells with no major detectable time delay, as well as the presence of connexin43 immune reactivity at the donor—host interface are compelling evidence that PSC grafts electrically couple to the recipient muscle via gap junctional channels, and further, that they develop a mature Ca^2+^ handling phenotype following stable engraftment. Identical results have been obtained following intracardiac transplantation of MHC-EGFP fetal ventricular cardiomyocytes in adult syngeneic hosts (Rubart et al., 2003).

**Figure 4:**
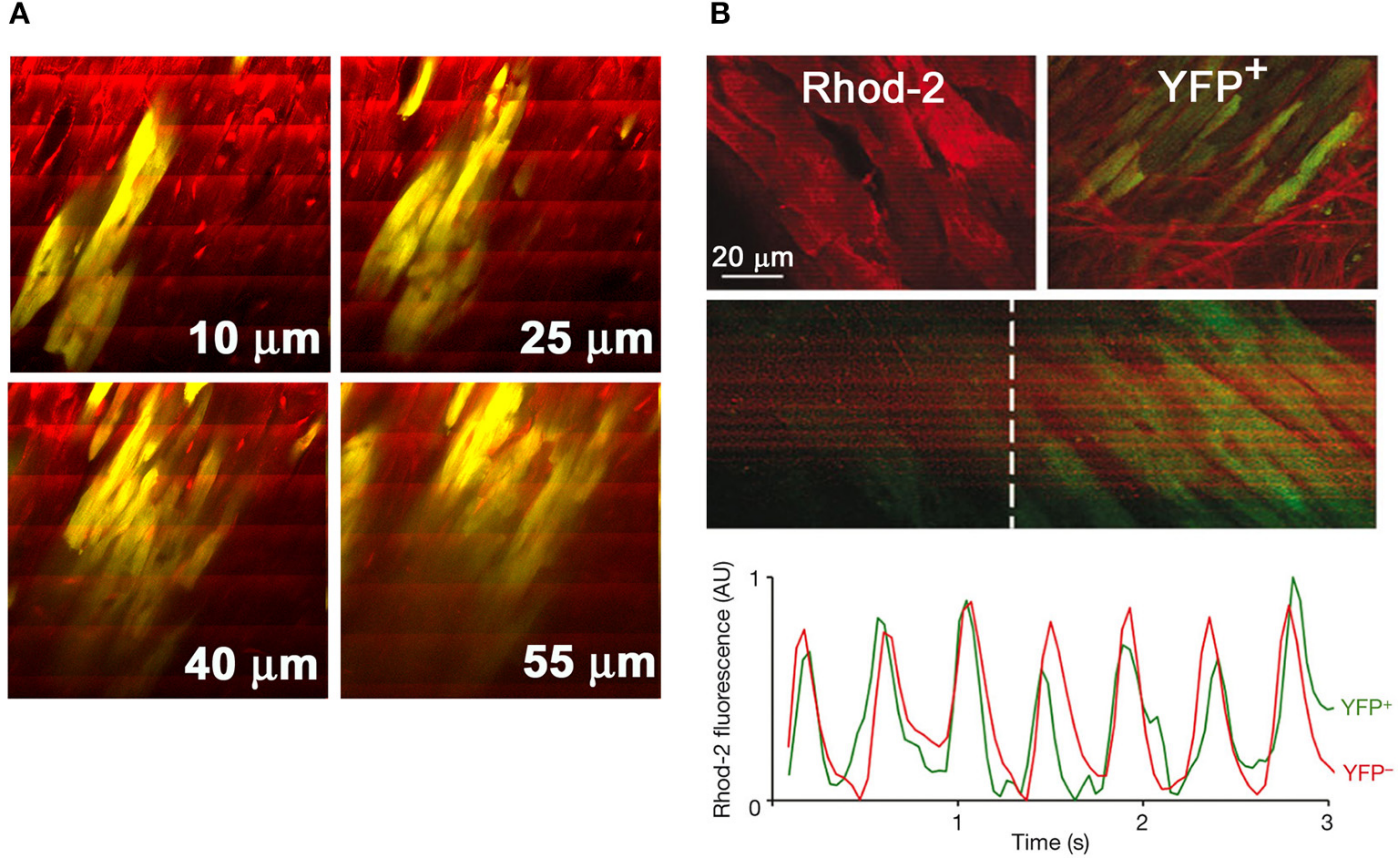
**TPLSM-based functional assessment of intracardiac cell transplants and cardiac stem cell-derived *de novo* cardiomyocytes *in situ*. (A)** Series of frame-mode images acquired from a rhod-2—loaded Langendorff-perfused mouse heart bearing a graft composed of EGFP-expressing fetal ventricular cardiomyocytes (yellow due to the overlap of red rhod-2 and green EGFP fluorescence). The heart was continuously paced at 2 Hz during image acquisition. Periodic increases in rhod-2 fluorescence intensity are visible as ripple-like wave fronts spanning the entire image width. Numbers in μm denote nominal distance along the *z*-axis from the epicardial surface. With permission from the American Heart Association. **(B)** Activated Wilm's tumor 1 (Wt1)-positive epicardial progenitors differentiate into functionally coupled cardiomyocytes in the chronically infarcted mouse heart. Transgenic hearts with conditional expression of yellow fluorescent protein (YFP) selectively in Wt1^+^ epicardial progenitor cells were subjected to TPLSM [Ca^2+^]_i_ imaging at 14 days following permanent coronary artery ligation. *X-Y* images illustrate rhod-2 fluorescence in YFP^−^ myocytes and in a neighboring cluster of YFP^+^ myocytes in the peri-infarct zone (upper two panels). [Ca^2+^]_i_ transients elicited by propagating action potentials are visible as band-like, periodic increases in rhod-2 fluorescence intensity in YFP^+^ myocytes (middle two panels), with kinetics indistinguishable from those of neighboring YFP^−^ cardiomyocytes (lower panel). From: Smart et al. (2011). With permission from Nature Publishing.

Given the improved penetration depth of two-photon vs. confocal microscopy in living tissue, TPLSM [Ca^2+^]_i_ imaging is also capable of functionally screening a large number of donor myocytes located up to several tens of microns below the epicardial surface in Langendorff-perfused mouse hearts. Figure 4A illustrates a series of frame-mode images that were acquired from a rhod-2 loaded mouse heart also carrying an EGFP-expressing, fetal ventricular myocyte graft. Action potential-evoked rhod-2 transients occur synchronously across donor and host cardiomyocytes during remote electrical pacing at 2 Hz, indicating that donor myocytes couple with each other as well as with host myocytes.

Paul Riley's group recently used TPLSM [Ca^2+^]_i_ imaging to probe functional integration of cardiac progenitor-derived *de novo* myocytes in the chronically infarcted mouse heart, as shown in Figure 4B (Smart et al., 2011). Their experiments utilized a transgenic mouse model in which expression of a yellow fluorescent protein (YFP) marked a population of epicardial progenitor-derived myocytes. TPLSM imaging of the infarct border zone in rhod-2—loaded hearts revealed the occurrence of action potential-evoked [Ca^2+^]_i_ transients in YFP^+^ myocytes in synchrony with their neighboring YFP^−^ myocytes, indicative of electrical coupling between the two cell types. In addition, the [Ca^2+^]_i_ transient kinetics in YFP^−^ and YFP^+^ cardiomyocytes were indistinguishable from each other.

Although dual photon excitation-induced fluorescent signals can clearly be recorded at tissue depths exceeding 100 μm (Helmchen and Denk, 2005; Ghouri et al., 2013; Kelly et al., 2013), more recent studies found a depth-dependent decrease in the axial resolution of two-photon fluorescence microscopy (Niesner et al., 2007; Scherschel et al., 2008; Young et al., 2011; Ghouri et al., 2013), due to spherical aberration in refractive index mismatched biological samples. The degradation in axial resolution has to be considered when interpreting two-photon microscopy images obtained from biological specimens in non-descanned mode, i.e., without a pinhole in the emission path to reject out-of-focus fluorescence. Use of deconvolution algorithms taking into account depth-dependent changes in spatial resolution may alleviate this problem (Niesner et al., 2007).

**Figure 5:**
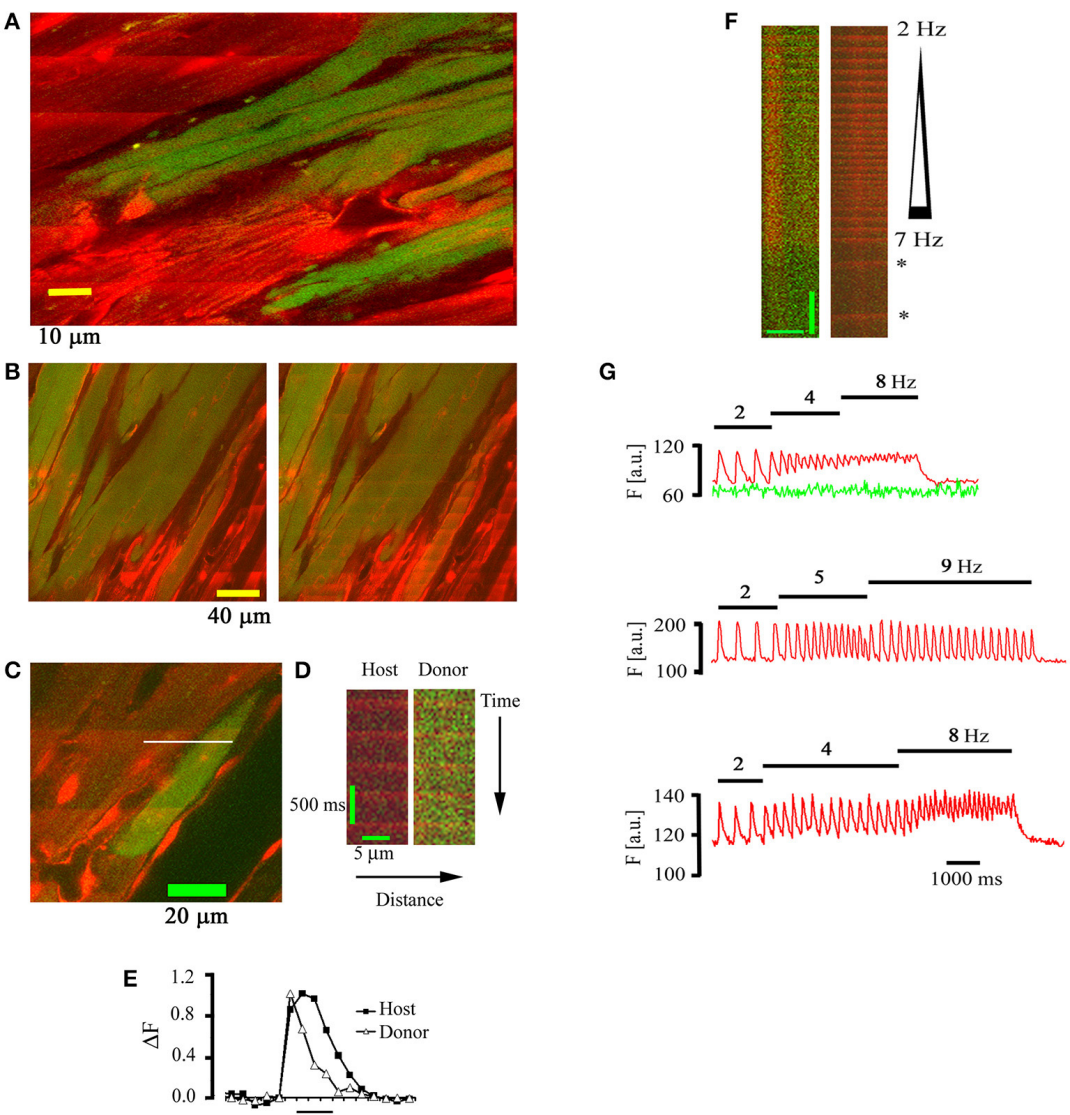
**Electrically evoked [Ca^2+^]_i_ transients in skeletal muscle grafts. (A)** Frame-mode image obtained at the graft-host border 18 days following intracardiac transplantation of EGFP-expressing skeletal myoblasts into a non-expressing mouse heart. The heart was continuously paced at a rate of 3 Hz at a remote point. Note the absence of electrically evoked rhod-2 transients in the skeletal muscle graft. **(B)** Frame-mode images obtained at the cardiac-skeletal muscle border obtained during spontaneous sinus rhythm (left panel) and electrical field stimulation (75 V, 1 ms, 4 Hz). **(C)** Frame mode image obtained from the skeletal-cardiac muscle junction in a rhod-2 loaded mouse heart 22 days following the transplantation of EGFP-expressing skeletal myoblasts. **(D)** Line-scan image obtained by repeatedly scanning along the white line in **(C)**. **(E)** Plots of normalized rhod-2 fluorescence as a function of time for the donor and host myocyte depicted in **(D)**. **(F)** Tetanic elevation of [Ca^2+^]_i_ in a functionally coupled donor-derived myocyte, but not in the juxtaposed host cardiomyocyte, during electrical field stimulation at increasing frequency. Asterisks denote [Ca^2+^]_i_ transients during spontaneous sinus rhythm. Scale bars: 10 μm horizontally, 1 s vertically. **(G)** Plots of spatially averaged rhod-2 (red tracing) and EGFP (green tracing) fluorescence intensities in a donor-derived (upper panel) and host (middle panel) myocyte *in situ* and in a skeletal myotube *in vitro* (lower panel) during incremental rates of electrical field stimulation (stimulation rates are denoted above each plot). With permission from the Journal of Clinical Investigation.

Intracardiac transplantation of autologous skeletal myoblasts has been shown to improve left ventricular performance in post myocardial infarct patients with congestive heart failure, albeit at an increased risk of experiencing ventricular tachyarrhythmias (Menasché et al., 2003). To investigate functional interactions between skeletal and cardiac muscle at the cellular scale *in situ*, hearts bearing EGFP-expressing skeletal muscle grafts were subjected to TPLSM [Ca^2+^]_i_ imaging as outlined above (Rubart et al., 2004b). Representative outcomes are illustrated in Figure 5. The vast majority of EGFP-expressing, i.e., donor-derived, myocytes were electrically isolated from the host myocardium, as evidenced by the absence of [Ca^2+^]_i_ transients in response to remote point electrical stimulation (Figure 5A). Electrical field stimulation of the intact heart readily evoked [Ca^2+^]_i_ transients in skeletal grafts, indicating that the absence of [Ca^2+^]_i_ responses resulted from a lack of action potential transmission from host to graft muscle rather than from a dysfunction of the depolarization-induced Ca^2+^ release mechanism in engrafted myotubes. A small number of donor-derived myocytes exclusively located at the graft-host border exhibited [Ca^2+^]_i_ transients in phase with those in the neighboring host cardiomyocytes (Figures 5C,D). Transients in donor-derived myocytes could exhibit different kinetics compared to their juxtaposed host myocytes, creating potentially arrhythmogenic spatial heterogeneity in Ca^2+^ handling. Intriguingly, these cells, but not their immediate cardiomyocyte neighbors, readily developed tetanic [Ca^2+^]_i_ elevations in response to electrical stimulation at incremental rates, compatible with a skeletal muscle-like Ca^2+^ handling phenotype (Figures 5F,G). Additional studies utilizing host and donor cell specific reporter transgenes and connexin43 immunohistology indicated that the coupled donor-derived myocytes resulted from spontaneous donor-host cell fusion events, giving rise to a hybrid phenotype exhibiting cardiomyocyte- (electrical coupling via gap junctions) and skeletal muscle (tetanus)–like features. Because intracardiac skeletal muscle grafts retain a voltage-activated Ca^2+^ release mechanism and skeletal myoblasts can be generated from autologous sources in large quantities for transplantation purposes, current experiments are testing the ability of connexin43-expressing skeletal muscle grafts to form a functional syncytium with the adult mouse myocardium, using TPLSM [Ca^2+^]_i_ imaging.

Overall, our results demonstrate the utility of our optical assay to monitor functional donor-host cell interactions at the cellular scale *in situ*. In addition to the two donor cell types presented here, we have successfully used TPLSM [Ca^2+^]_i_ imaging to interrogate the functional fate of transplanted hematopoetic stem cells (Scherschel et al., 2008), cardiospheres (Shenje et al., 2008), as well as fetal atrial and ventricular cardiomyocytes (Rubart et al., 2003). In addition to its ability to quantitatively assess, at the level of the individual cell, the extent to which donor cells functionally integrate with the host myocardium following their intracardiac transplantation, the assay is also capable of examining potential adverse effects (e.g., arrhythmogenic calcium signaling) imparted by cellular transplants onto the host myocardium. Finally, it can be used as a method to guide interventions aimed at enhancing the low long-term seeding efficiency of cellular transplants.

A possible limitation of the approach presented here is the use of mouse hearts. Human embryonic stem cell-derived cardiomyocytes have recently been shown to stably and functionally couple with the host myocardium in infarcted hearts of guinea pigs and non-human primates, alleviating postinfarction left ventricular dysfunction and susceptibility to ventricular arrhythmias (Shiba et al., 2012; Chong et al., 2014). Prior work by others (van Laake et al., 2008) suggest that the rapid heart rates of mice (ca. 600 beats per minute) may prevent functional coupling of human embryonic stem cell-cardiomyocytes with murine host myocardium. Accordingly, to be able to extend the advantages of our assay to the assessment of human embryonic stem cell-cardiomyocyte grafts, its adaptability to larger species with lower heart rates should be examined. Within this context, the observation by others that TPLSM [Ca^2+^]_i_ or V_m_ imaging can be applied to the rabbit heart is promising (Ghouri et al., 2013; Kelly et al., 2013).

## Assessment of spatiotemporal dispersion of electrical and Ca^2+^ signals in langendorff-perfused hearts using laser scanning microscopy

**Figure 6:**
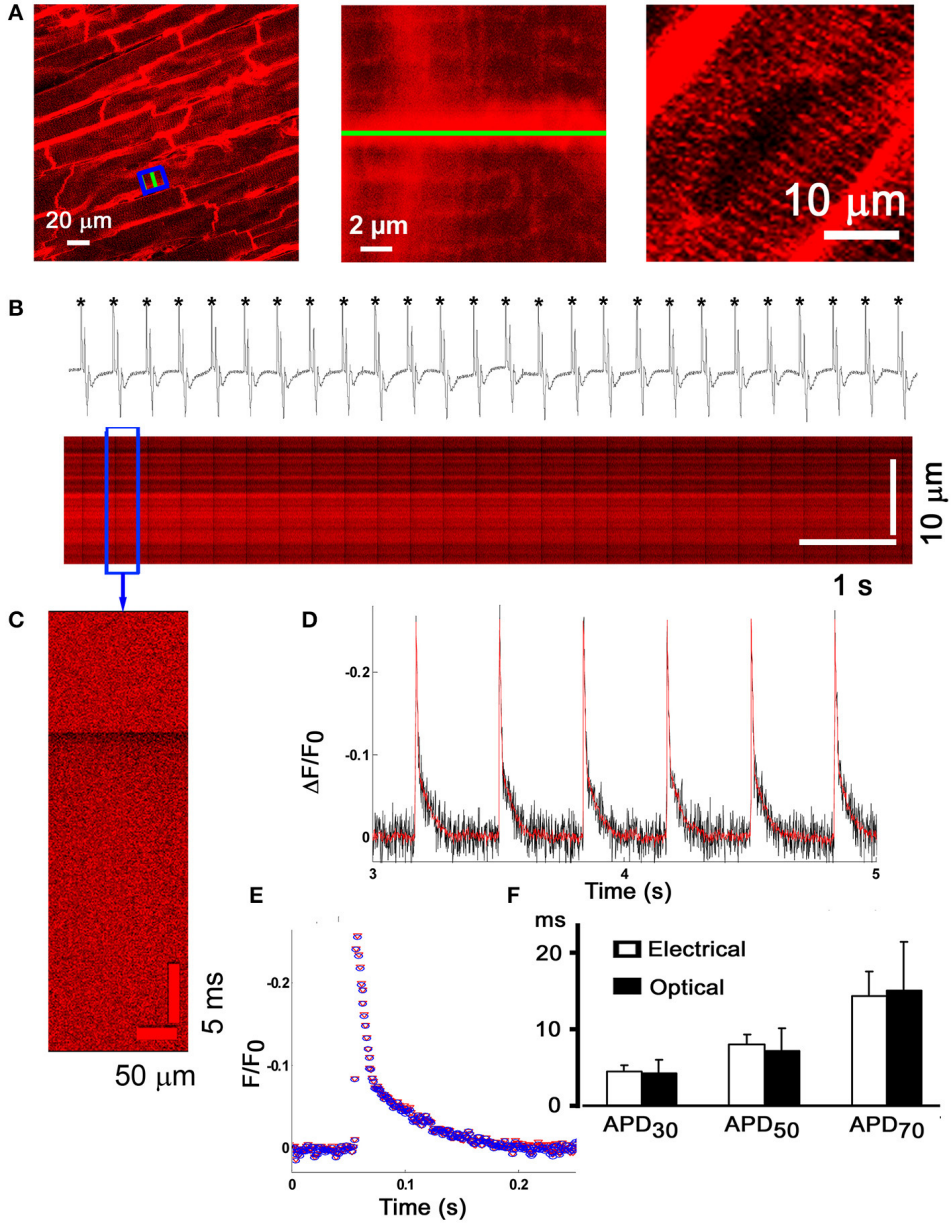
**Micron-scale imaging of transmembrane voltage changes in the Langendorff-perfused mouse heart. (A)** Frame-mode confocal image taken from the anterior left ventricular epicardial layer of a Langendorff-perfused mouse heart loaded with the fast-response voltage-sensitive dye Annine-6plus (left panel). The dye was excited at 488 nm and fluorescence emission was measured at >560 nm. Cytochalasin D (50 μmol/L) and ryanodine (1 μmol/L) were present in the coronary perfusate to suppress motion. Middle panel, magnified view of the boxed area in **(A)**, revealing dye accumulation in the t-tubular membranes (thin lines). Right panel, no staining of the nuclear envelope was detectable more than 3 h after dye loading. **(B)** Line-scan mode image acquisition of action potential-evoked changes in Annine-6plus fluorescence intensity. The green line in **(A)** (left and middle panel) was repetitively scanned at a rate of 1 kHz and successive lines were stacked horizontally. Periodic, sharp decreases in Annine-6plus fluorescence intensity occur in phase with the QRS complex in the simultanesously recorded volume-conducted ECG, indicating that they correspond to transmembrane voltage changes in response to propagating action potentials. **(C)** Expanded view of changes in normalized Annine-6plus fluorescence intensity during a single evoked action potential. **(D)** Plots of spatially averaged changes in normalized Annine-6plus fluorescence intensity derived from the line-scan in **(B)**. Red line corresponds to the filtered signal (ref). **(E)** Superimposition of the ensemble average of all consecutive action potentials in the line-scan in **(B)** and the filtered signal of a single optical action potential. **(F)** Bar graphs comparing optically and electrically measured action potential durations. There were no significant differences for any of the three time points assessed. With permission from the Biophysical Society.

To be able to directly assess electrical activity on a microscopic scale *in situ*, we previously developed an optical assay using laser confocal scanning imaging in conjunction with the fast-response, voltage-sensitive dye Annine-6plus (Bu et al., 2009). Annine-6plus reduces its fluorescence during membrane depolarization and vice versa. Langendorff-perfused mouse hearts were loaded with this water soluble indicator, perfused with Tyrode's solution containing cytochalasin D (50 μmol/L) and ryanodine (1 μmol/L) to suppress motion, and subjected to laser scanning microscopy. Confocal images obtained from the left anterior epicardial layer reveal thick lines of high fluorescence intensity corresponding to dye staining of the surface membranes in adjacent cardiomyocytes, whereas thin lines running perpendicular to the outer membranes represent t-tubular membranes (middle panel in Figure 6A). No staining of the nuclear envelope or other internal lipid bilayer membranes was discernible at >3 h after due loading (right panel in Figure 6A), supporting the notion that the dye stably and selectively stains the outer membrane of cardiomyocytes. Line-scan mode images (Figures 6B,C) display periodic decreases in dye fluorescence occurring in phase with the electrocardiographic QRS complexes during electrical point stimulation at 3 Hz. From the line scan images, the spatially averaged signals were obtained and plotted as a function of time. The ensemble average of all consecutive transients in a line-scan image (~25) and the filtered signal of individual transients exhibited similarly high signal-to-noise ratios (Figures 6D,E). The time course of fluorescence recovery during an optical action potential paralleled the time course of the electrically measured action potential repolarization (Figure 6F), supporting the utility of the optical assay to resolve, on a subcellular scale, changes in transmembrane potential typically occurring during the downstroke of a cardiomyocyte action potential. Annine-6plus exhibits a linear response with a voltage-sensitivity of 0.29 ± 0.01%/mV.

**Figure 7:**
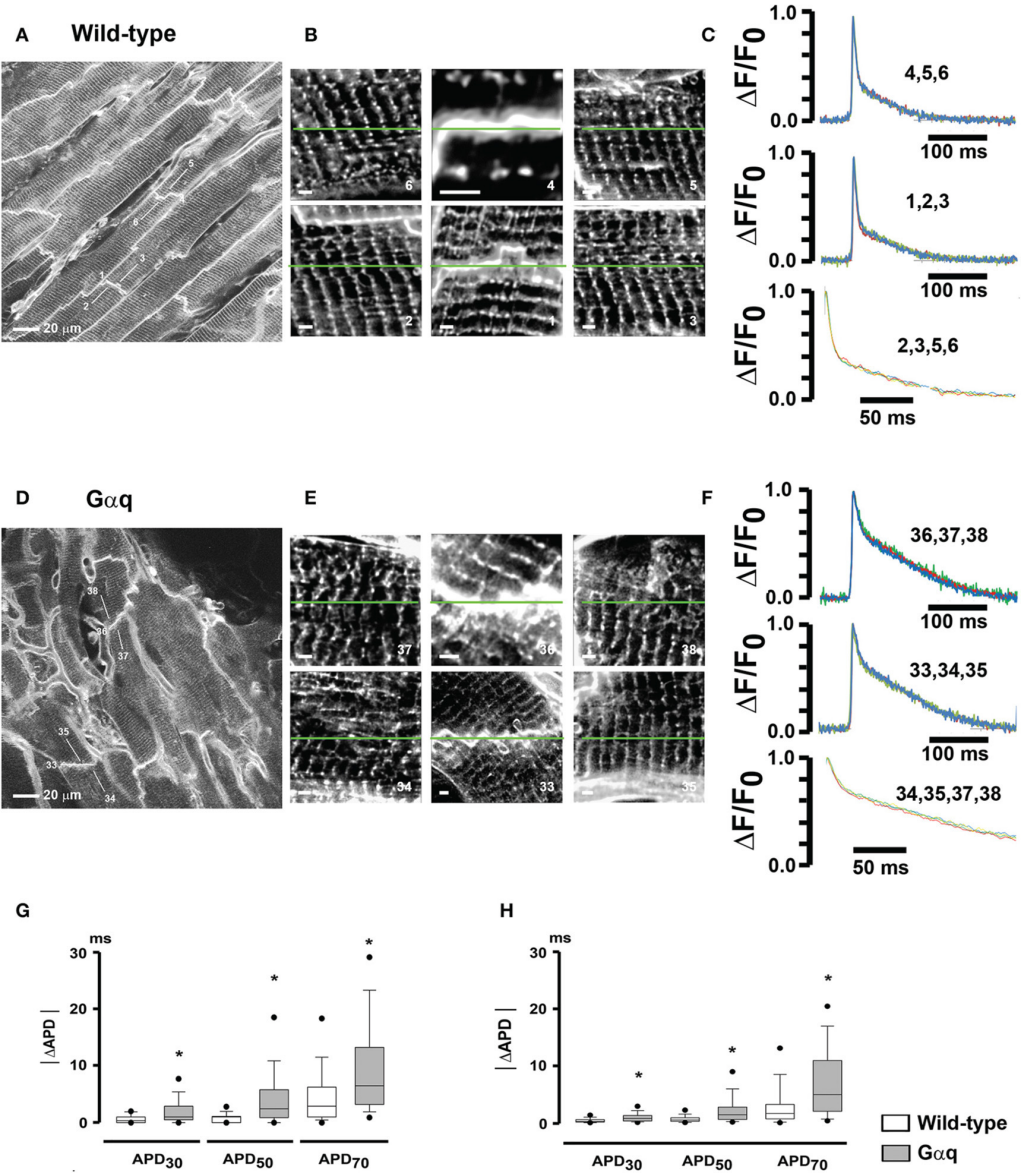
**Increased micro-heterogeneity in cardiomyocyte repolarization in hearts with pathological hypertrophy. (A,D)** full-frame mode confocal images obtained from a wild-type heart **(A)** and a transgenic heart with cardiomyocyte-restricted overexpression of Gαq **(D)** loaded with Annine-6plus. Numbered white lines denote positions of line-scan mode acquisitions for action potential recording. **(B,E)** High-zoom 2D scans of areas centered in the correspondingly numbered line-scan positions in **(A,D)**. Each image is composed of a square-shaped region with side length equal to the length of the respective action potential scan line. White scale bars, 5 μm. **(C,F)** Overlays of normalized optical action potentials recorded along the green lines in **(B,E)**. Numbers above the trace refer to the numbers of the scan lines in **(B,E)**. Stimulation rate was 3 Hz. Lower traces show overlays of 200-ms repolarization segments starting from the peak. Traces were filtered using moving average. **(G)** Box-and-whisker plots of absolute APD differences (|ΔAPD|) between individual cardiomyocytes within the same 225 × 225 μm^2^ acquired image frames. ^*^P ≤ 0.001 vs. wild-type. **(H)** Electrically measured |ΔAPD| between individual cardiomyocytes within small microscopic (~500 μm in diameter) locations in the anterior left ventricular epicardial layers of wild-type and Gαq transgenic hearts. ^•^*P* ≤ 0.005 vs. wild-type. With permission from Journal of Molecular and Cellular Cardiology.

We subsequently employed our optical assay to examine, on a microscopic scale, left ventricular action potential remodeling in a mouse model of pathological hypertrophy, produced by cardiac-restricted Gαq overexpression (Tao et al., 2013). Confocal images obtained from randomly selected 225 × 225 μm^2^ regions across the anterior LV epicardium of immobilized (50 μM cytochalasin D and 1 μM ryanodine), Annine-6plus—loaded hearts revealed orderly T-tubule arrangements in the wild-type myocytes *in situ*, whereas Gαq-overexpressing cells exhibited spatially non-uniform t-tubular disorganization with patchy loss or growth of t-tubules (Figures 7A,B,D,E). Shape and time course of repolarization appeared to exhibit small variations among individual wild-type and Gαq cardiomyocytes located within the same microscopic region (Figures 7C,F). Calculation of pairwise absolute APD differences (|ΔAPD|) between individual *in situ* cardiomyocytes within each region revealed that for each time point of repolarization |ΔAPD| values on average were significantly more pronounced in Gαq compared to wild-type hearts, suggesting an increase in microscopic dispersion of repolarization within the LV epicardium (Figure 7G). Results of these optical measurements were recapitulated in electrical recordings of transmembrane action potentials across the anterior LV epicardial layer (Figure 7H). Additional structural analyses demonstrated similarly low levels of interstitial collagen deposition as well as equal densities and distributions of connexin43 in the LV epicardium of wild-type and transgenic mice. Together, these results indicate that pathological LV hypertrophy associated with cardiac Gαq overexpression results in heterogeneous alterations of t-tubular micro-architecture and spatially non-uniform action potential prolongation in the LV epicardium, increasing APD dispersion on a microscopic scale. Further, the data support the notion that small portions of electrically well connected cardiac tissue can maintain small, yet significant, repolarization gradients, possibly through heterogeneity in the intrinsic repolarization properties of individual cardiomyocytes. These findings are in agreement with those by others reporting small-scale APD differences in the hypertrophied cardiac muscle in the absence of significant changes in the spatial profile of electrotonic coupling (Keung and Aronson, 1981), and in human hearts exhibiting steep (27 ms/mm) repolarization gradients occurring over short distances (Glukhov et al., 2010).

Overall, we have shown that rapid line scanning confocal microscopy in conjunction with a voltage-sensitive dye enables, on a sub- to multi-cellular scale *in situ*, measurements of transmembrane voltage changes typically occurring during repolarization of the cardiac action potential. With improvements in temporal resolution of the scanning system as well as in the voltage-sensitivity of the dye, it should become possible to resolve fast propagation of the action potential along the t-tubule system in cardiomyocytes. Specifically, it will be interesting to examine whether structural t-tubule remodeling typically seen in the diseased heart, including increased radial length and/or tortuosity of individual t-tubules in hypertrophied myocytes, gives rise to impaired action potential propagation across the t-tubular network, resulting in increased asynchrony in activation of SR Ca^2+^ release sites and, thus, contractile dysfunction.

**Figure 8:**
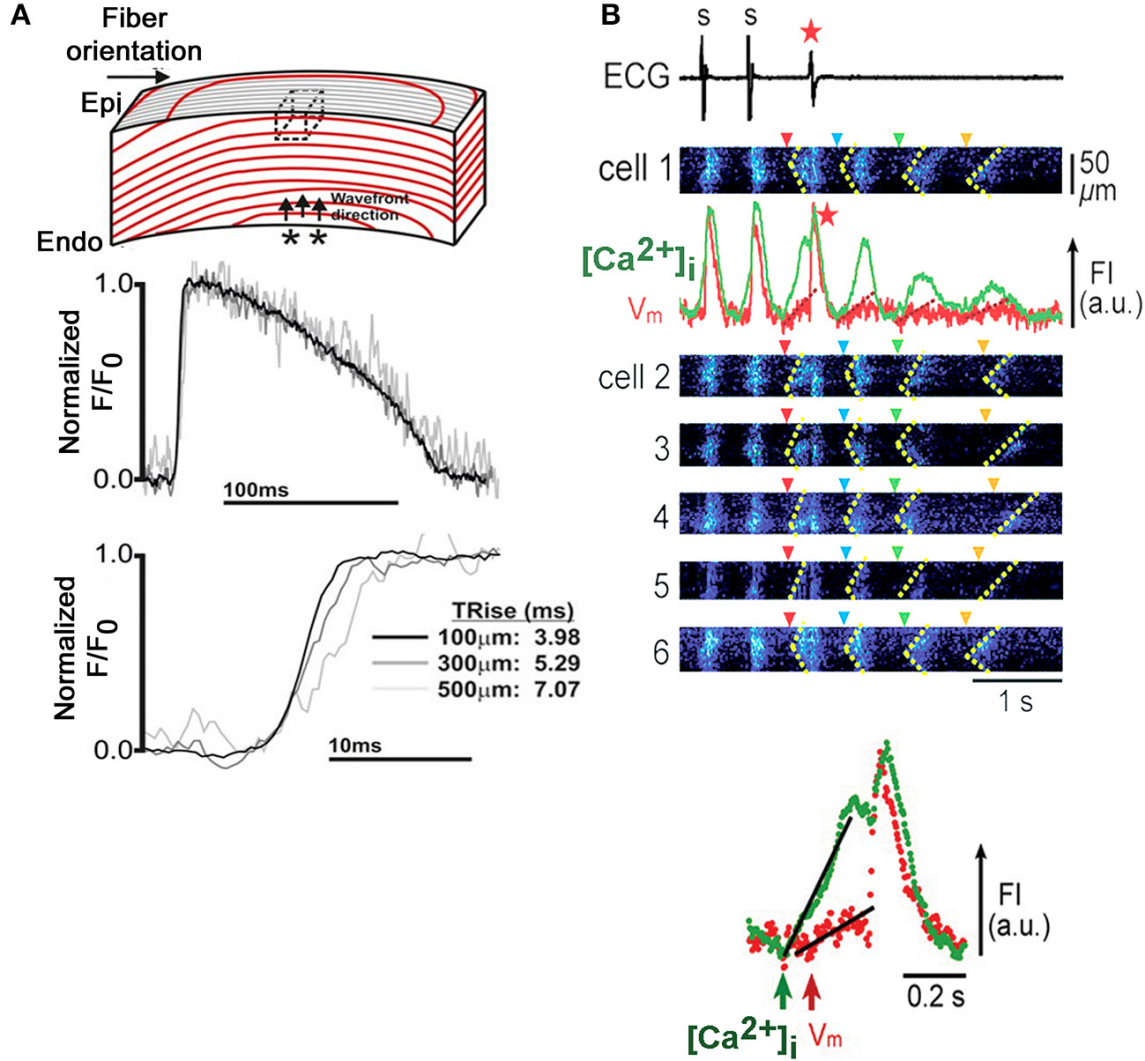
**Micron-scale V_m_ and [Ca^2+^]_i_ imaging in Langendorff-perfused rabbit and rat hearts. (A)** Depth-dependent increase in action potential upstroke rise time in an isolated rabbit heart. An isolated perfused rabbit heart was loaded with the ratiometric, voltage-sensitive dye di-4-ANEPPS and subjected to TPLSM. Immobilization was achieved by combined administration of blebbistatin (10 μmol/L) and 2,3-butanedione monoxime (10 mmol/L). Action potential-related changes in dye florescence (F) were recorded in line-scan mode from muscle layers at 100, 300, and 500 μm below the epicardial surface during endo- to epicardial wave front propagation and normalized to baseline fluorescence (F_o_). Upstroke rise time progressively increased with increasing tissue depth. Dotted box in the upper panel denotes the tissue volume from which fluorescence could be recorded in two-photon excitation mode. From Kelly et al. (2013). With permission from the American Heart Association. **(B)** Triggered activity mediated by Ca^2+^ waves in an intact rat heart. Upper panel: Synchronous emergence of Ca^2+^ waves (dotted lines in *x-t* images) in individual cells (1 through 6) accompanies a single event of triggered activity (red asterisk), followed by Ca^2+^ oscillations (green trace), with concomitant membrane fluctuations (red trace) showing a gradual reduction in amplitude. Only the first Ca^2+^ wave (red arrowhead), not the following three (blue, green and yellow arrowheads), triggers a propagating impulse. Fluorescence intensities of the calcium- and voltage-sensitive dyes fluo-4 and RH237, respectively, were measured in adjacent subepicardial ventricular myocytes within a Langendorff-perfused rat heart with dual-view, rapid scanning confocal microscopy. The triggered activity was induced by 2-Hz pacing under low K^+^ (2.4 mmol/L) perfusion and isoproterenol (3 nmol/L). Lower panel: Simultaneous V_m_ and intracellular Ca^2+^ recordings during the triggered beat demarcated by the red arrowhead in the upper panel at an expanded time scale demonstrate that the increase in [Ca^2+^]_i_ precedes membrane depolarization. FI, fluorescence intensity; a.u., arbitrary units (Modified from Fujiwara et al., 2008. With permission from the American Heart Association).

Recently, Godfrey Smith's laboratory was able to implement TPLSM V_m_ imaging in the isolated perfused rabbit heart, using the ratiometric, voltage-sensitive dye di-4-ANEPPS (Kelly et al., 2013). In the presence of the excitation-contraction uncoupler blebbistatin and 2,3-butanedione monoxime, they were able to resolve action potentials up to a depth of 500 μm from the left ventricular epicardial surface, revealing a progressive increase in action potential upstroke rise time with increasing depth during endo- to epicardial wave front propagation (Figure 8A). Conduction velocity progressively decreased with distance from the epicardial surface. Although structural details could not be resolved beyond an imaging depth of 300 μm, spatially limiting information about structure-function relationships, the results demonstrate the feasibility of functional TPLSM imaging in hearts much larger than rodent hearts, enhancing translatability of the information gained into the clinical arena.

Chen et al. used *in situ* confocal V_m_ and [Ca^2+^]_i_ imaging to investigate cardiac electrical signaling in a murine catecholaminergic polymorphic ventricular tachycardia model of type 2 ryanodine receptor[RyR]^*R*4496*C*+/−^ mutation (Chen et al., 2012). They found that RyR^4496*C*+/−^ hearts exhibited a high temporal variability of Ca^2+^ release during adrenergic stimulation, which was synchronized among individual, neighboring cardiomyocytes, and correlated with the occurrence of ventricular tachycardia. Intriguingly, a similar pattern of action potential variability, which was also synchronized among neighboring myocytes, was revealed in the adrenergically stressed intact RyR^4496*C*+/−^ heart but not in isolated RyR^4496*C*+/−^ myocytes. Based on these observations it was speculated that the Ca^2+^ release variability in the ventricular myocardium arises as a consequence of altered electrical activation during adrenergic stress, rather than from the failure of the Ca^2+^ release response to propagating action potentials. However, since the electrical and Ca^2+^ signals were recorded in separate experiments, this hypothesis awaits direct confirmation via simultaneous V_m_ and [Ca^2+^]_i_ *in situ* imaging on a microscopic scale.

The ability of simultaneous V_m_ and [Ca^2+^]_i_ confocal imaging *in situ* was previously shown in Langendorff-perfused rat hearts as illustrated in Figure 8B (Fujiwara et al., 2008). The heart was loaded with the fluorescent calcium and voltage sensors fluo-3 and RH237, respectively, and immobilized with 50 μmol/L cytochalasin D. Under experimental conditions favoring the development of bursts of spontaneous Ca^2+^ waves, it was possible to directly demonstrate that Ca^2+^ waves can evoke arrhyhmogenic membrane voltage changes when they emerge synchronously among multiple, neighboring cardiomyocytes, in agreement with a more recent dual V_m_-[Ca^2+^]_i_ optical mapping study in isolated rabbit hearts (Myles et al., 2012).

Collectively, techniques have been and are being developed to monitor Ca^2+^ and electrical signals on a microscopic scale in intact hearts, continuously enhancing our understating of the role of abnormal Ca^2+^ handling on a (sub)cellular level in the genesis of triggered arrhythmias in the heart. Improvements in (i) penetration depth of two-photon excitation microscopy via e.g., use of dyes that can be excited at wavelengths >1 μm, (ii) detector sensitivity, and (iii) speed of image acquisition will aid in accelerating discovery.

## Conclusions

Single- or dual-photon laser scanning microscopy can provide important insights into electrophysiological properties and Ca^2+^ regulation of individual *in situ* cardiomyocytes in immobilized, Langendorff-perfused mouse heart. A current limitation of the methods presented here is the use of pharmacological excitation-contraction blockers, i.e., cytochalasin D alone or in combination with ryanodine, which can modify the Ca^2+^ handling and electrical properties of the cells under study. Blebbistatin is a possible alternative, but, like cytochalasin D, it only insufficiently suppresses motion during micron scale imaging (Ghouri et al., 2013; Kelly et al., 2013; unpublished observation), requiring additional means of motion suppression.

Improvements in the voltage-sensitivity of fluorescent dyes, e.g., via shifted excitation and emission ratioing (Manno et al., 2013), in conjunction with faster acquisition techniques can further enhance the ability of laser scanning microscopy to spatially and temporally resolve fast and/or low-amplitude changes in transmembrane potential such as those occurring during the action potential upstroke and membrane afterdepolarizations, respectively, in cardiomyocytes *in situ*.

Finally, refinements of protein-based voltage- and calcium-sensors are expected to improve their sensitivity, enabling signal detection even with low light systems like confocal scanners. Targeting expression of these sensors to cardiac cells of interest (provided an appropriate cell type-specific promoter is available) should provide valuable insights into electrophysiology and Ca^2+^ regulation in intact heart muscle.

### Conflict of interest statement

The authors declare that the research was conducted in the absence of any commercial or financial relationships that could be construed as a potential conflict of interest.

## Abbreviations

TPLSM: two-photon laser scanning microscopy
PSC: parthenogenetic stem cell
MHC: alpha myosin heavy chain
EGFP: enhanced green fluorescent protein.
